# Analysis of the Langat Virus Genome in Persistent Infection of an *Ixodes scapularis* Cell Line

**DOI:** 10.3390/v8090252

**Published:** 2016-09-10

**Authors:** Luwanika Mlera, Wessam Melik, Danielle K. Offerdahl, Eric Dahlstrom, Stephen F. Porcella, Marshall E. Bloom

**Affiliations:** 1 Biology of Vector-Borne Viruses Section, Laboratory of Virology, National Institutes of Health, Hamilton, MT 59840, USA; Luwanika.Mlera@nih.gov (L.M.); Wessam.Melik@oru.se (W.M.); offerdahld@niaid.nih.gov (D.K.O.); 2 Genomics Unit, Research Technologies Branch, Hamilton, MT 59840, USA; eric.dahlstrom@nih.gov (E.D.); SPORCELLA@niaid.nih.gov (S.F.P.)

**Keywords:** Langat virus genome, tick-borne flavivirus, persistent infection, *Ixodes scapularis*, ISE6 cells, deep-sequencing

## Abstract

Tick-borne flaviviruses (TBFVs) cause a broad spectrum of disease manifestations ranging from asymptomatic to mild febrile illness and life threatening encephalitis. These single-stranded positive-sense (ss(+)) RNA viruses are naturally maintained in a persistent infection of ixodid ticks and small-medium sized mammals. The development of cell lines from the ixodid ticks has provided a valuable surrogate system for studying the biology of TBFVs in vitro. When we infected ISE6 cells, an *Ixodes scapularis* embryonic cell line, with Langat virus (LGTV) we observed that the infection proceeded directly into persistence without any cytopathic effect. Analysis of the viral genome at selected time points showed that no defective genomes were generated during LGTV persistence by 10 weeks of cell passage. This was in contrast to LGTV persistence in 293T cells in which defective viral genomes are detectable by five weeks of serial cell passage. We identified two synonymous nucleotide changes i.e., 1893A→C (29% of 5978 reads at 12 h post infection (hpi)) and 2284T→A (34% of 4191 reads at 12 hpi) in the region encoding for the viral protein E. These results suggested that the mechanisms supporting LGTV persistence are different between tick and mammalian cells.

The tick-borne flaviviruses (TBFVs) are associated with a variety of clinical diseases in humans, ranging from asymptomatic to mild febrile illness or severe, sometimes fatal meningoencephalitis or hemorrhagic fever [1,2,3,4,5]. In spite of the fact that there is an effective vaccine [6,7], there are 10,000–15,000 TBFV infections each year with mortalities as high as 20%, depending on the particular virus [2,8]. Although much of the TBFV morbidity and mortality results from acute infections, there is increasing evidence that chronic or persistent infection may lead to long-term illness and sequelae [9].

TBFV infection typically results from the bite of an infected ixodid or hard-bodied tick, and because the viruses have a global distribution, the principal vector species varies from region to region. For example, the principal vectors for Powassan virus in North America are *Ixodes scapularis* and *Ixodes cookei*, whereas *Haemaphysalis longicornis* transmits the virus in East Asia [10]. However, the Alkhurma virus utilizes a soft-bodied or argasid tick, *Ornithodoros savignyi*, as a vector, so perhaps TBFV vector competence is broader than generally appreciated [11]. Nevertheless, once the ticks are infected, the virus persists across the various life stages, and can be passed transovarially to progeny [12]. Co-feeding of infected and uninfected ticks on the same host animal demonstrates that horizontal transmission of the virus among ticks also occurs [2,13,14,15].

Clearly, the interactions of TBFV with invertebrate hosts present a complex vector-pathogen relationship, and the biology of virus persistence is an important facet. The study of these relationships has been greatly aided by the development of cell lines derived from ixodid ticks [16,17]. We have recently initiated studies to characterize viral persistence and determine its role in the biology of TBFV infections [18,19]. Infection of mammalian cells in vitro with TBFVs and some encephalitic mosquito-borne flaviviruses leads to an acute lytic crisis mediated by apoptosis [18,20,21]. However, a persistent infection is initiated in the few surviving cells and persistence is maintained indefinitely [19,22,23,24,25]. Using extensive unbiased next-generation sequencing, we demonstrated that defective genomes, which would be packaged to become defective interfering particles (DIPs) are not present at the initiation of viral persistence in mammalian cell lines, but are a prominent feature during the maintenance of viral persistence [19]. In marked contrast to mammalian cells, infection of cell lines derived from *Ixodes*, *Boophilus*, *Hyaloma*, *Ornithodoros* or *Rhipicephalus* tick species does not lead to apparent cell death or obvious cytopathological changes [23,26]. Furthermore, we showed that infection of ISE6 cells derived from *Ixodes scapularis* embryos [27] with a TBFV leads directly to viral persistence [23]. However, in that previous work we did not examine TBFV genomes in detail. Given the results of our studies on TBFV persistence in mammalian cells, we wanted to evaluate the viral genome stability of persistent TBFV infection in ISE6 cells with the same methodology. Therefore, in this publication, we have established a model system for TBFV persistence in ISE6 cells and have used unbiased deep-sequencing to investigate potential genomic evolution and alterations. During persistent TBFV infection of these cells, the TBFV genome was remarkably stable and no evidence of truncated genomes or DIPs was observed.

In order to do these studies, we infected 1.5 × 10^6^ ISE6 cells in 25 cm^2^ CellStar^®^ flasks (Greiner Bio-One, Kremsmünster, Austria) with Langat TP21 virus [28] derived from a full length molecular clone [19] at a multiplicity of infection (MOI) of 5 for 1 h at 37 °C with rocking. The infecting medium was removed and cells were washed three times with phosphate-buffered saline (PBS) and maintained in a L-15C300 medium supplemented with 5% tryptose phosphate broth, 5% fetal bovine serum (FBS) (Invitrogen; Life Technologies, Carlsbad, CA, USA), and 0.1% bovine lipoprotein concentrate (MP Biomedicals, Santa Ana, CA, USA) at 34 °C. Cultures were studied at selected time points after infection.

Following infection, the infected ISE6 cells were observed closely for evidence of cytopathology or a lytic crisis, as was noted in our previous studies on mammalian cells [19]. At no time was evidence of cytopathology or crisis observed, a result consistent with our earlier studies [23].

Immunofluorescence was used to evaluate the extent of Langat virus (LGTV) infection in the ISE6 cultures. 10^5^ ISE6 cells in 4-well Labtek chamber slides (Nunc^®^, Sigma-Aldrich, Atlanta, GA, USA) were infected at a MOI of 5, and prepared for immunofluorescent microscopy at 12, 96, and 1680 h post infection (hpi). At each time point, cells were washed twice with PBS, fixed with 4% paraformaldehyde, probed with a mouse monoclonal anti-E (11H12) antibody (a kind gift from Dr. Connie Schmaljohn, USAMRID, Fort Detrick, Frederick, MD, USA) and counterstained with 4',6-diamidino-2-phenylindole (DAPI). Examination of these preparations revealed that few cells were infected at 12 hpi. However, a higher number of cells were infected at 96 hpi as shown by positive staining for the LGTV E protein (Figure 1A). Furthermore, the fraction of ISE6 cells positive for E appeared to remain stable out to 1680 h (Figure 1A). These results indicated that most cells in the cultures were expressing LGTV proteins by 96 hpi and maintained expression for an extended period.

In order to confirm that E protein expression corresponded to a persistent infection, we determined the course of LGTV titer and genome copies. Supernatants were harvested at 12, 48, 96, 336 and 1680 hpi for virus titration using an immunofocus assay as described before [19,23]. Virus titer peaked to 2.0 × 10^5^ ffu/mL at 96 hpi. At two weeks post infection (336 hpi), virus titer declined to 5.1 × 10^3^ ffu/mL, but showed a modest increase to 3.7 × 10^4^ ffu/mL at 1680 hpi (Figure 1B). Thus, a persistent infection had been initiated and was maintained.

To determine LGTV genome copy numbers, total cellular RNA was extracted at 12, 48, 96, 336 and 1680 hpi using an RNeasy kit (Qiagen, Los Angeles, CA, USA) as per the manufacturer’s instructions. Complementary DNA (cDNA) was synthesized from 1 µg of total RNA using a VILO cDNA kit (Invitrogen) according to the manufacturer’s instructions. 2 µL of the cDNA synthesis reaction was added to a quantitative PCR (qPCR) reaction mix containing LGTV-specific primers/probe (forward primer: GGATTGTTGCCCAGGATTCTC; probe: FAM-CATTGGCACCGGCCTACGCT-NFQ; and reverse primer: TTCCAGGTGGGTGCATCTC), IX Platinum qPCR Supermix UDG with Rox (Invitrogen, Life Technologies, Carlsbad, CA, USA). The qPCR assay was performed using a 7900HT fast real time PCR system (Applied Biosystems, Foster City, CA, USA).

The number of genome copies showed a similar pattern, but was several logs higher, again confirming persistent infection. Interestingly, we noted a decline in viral titer at 336 hpi that was associated with a corresponding dip in genome copy numbers (Figure 1C), suggesting that the replication rate was low at this time point. The 2-week time point corresponds to the time at which we normally split the cells, and this could have contributed to the lower rate of virus replication. Overall, these titers were comparable to those in our previous observations in ISE6 cells [23], as well as studies with tick-borne encephalitis virus (TBEV) in IDE2 cells, also derived from *Ixodes scapularis* [26], suggesting that the replication kinetics of these viruses was similar in cells from this tick species.

As mentioned, our recent studies in HEK 293T cells show that DIPs were not present at the initiation of persistent infection, but were a feature once persistence was established [19]. Consequently, we were curious to see if DIPs were present in persistently infected ISE6 cells. Therefore, we deep-sequenced the LGTV genome extracted from total cellular RNA on a HiSeq 2500 sequencer (Illumina, San Diego, CA, USA) as described before [19]. The sequence reads were aligned and visualized with Integrated Genomics Viewer software (version 2.2.10, Broad Institute, Cambridge, MA, USA) [29,30], and 253,809 sequence read pairs were aligned at 12 hpi resulting in a depth of coverage of 2300-fold. 2,884,383 sequence pairs were obtained at 96 hpi to achieve a sequencing depth of 26,000-fold. At 1680 hpi, we obtained 881,289 sequence pairs to achieve an average depth of coverage of 8000-fold. Interestingly, analysis of the LGTV genome alignments failed to identify truncated genomes at any of these time points (Figure 2). This contrasted with our observations of 293T cells, in which truncations could be detected as early as 5 weeks of passaging persistently infected cells [19]. These results suggested that DIPs are not generated during persistent TBFV infection of ISE6 cells.

We also compared the sequences at 12, 96 and 1680 hpi for any nucleotide sequence changes to an LGTV reference sequence (GenBank accession No. EU790644). The same nucleotide sequence changes, that we attributed to the rescue of LGTV in Vero cells [19], were also observed in the viral genome at all time-points studied. However, we identified two synonymous nucleotide changes i.e., 1893A→C (29% of 5978 reads at 12 hpi) and 2284T→A (34% of 4191 reads at 12 hpi) in the region encoding for the viral protein E. Interestingly, the synonymous nucleotide change 4299C→T in the region encoding for NS2B, which we detected in late LGTV persistence in 293T cells [19], was also detected only at 1680 hpi. The significance of this nucleotide change during viral persistence is unclear.

In summary, LGTV readily initiated a persistent infection in ISE6 cells with no evidence of an acute lytic phase. In contrast to mammalian cells, viral persistence was not associated with DIPs. These results suggested that the mechanisms supporting viral persistence may differ between tick and mammalian cells.

## Figures and Tables

**Figure 1:**
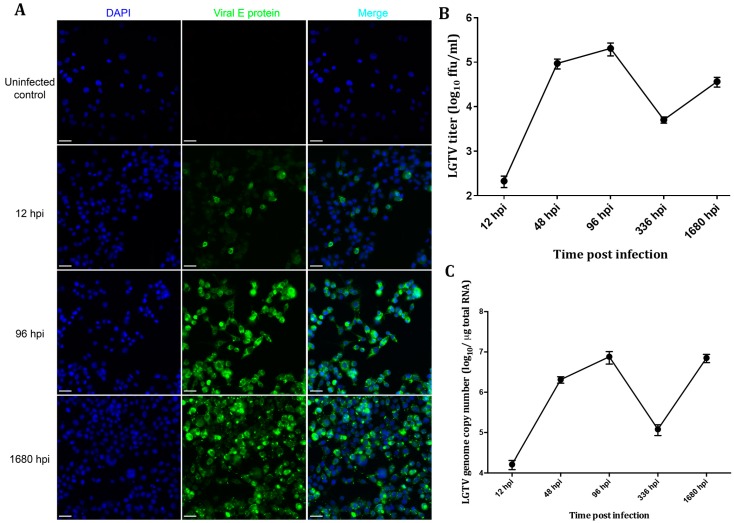
Langat virus (LGTV) replication kinetics in ISE6 cells. (**A**) Detection of the expression of LGTV E protein by confocal microscopy. Few cells were infected at 12 h post infection (hpi) as indicated by viral E protein staining in a low number of cells, but almost all of the cells were infected at 96 and 1680 hpi. The scale bar represents 10 µm; (**B**) LGTV TP21 titer obtained by an immunofocus assay using an anti-E antibody; (**C**) LGTV RNA genome copy numbers measured by quantitative PCR (qPCR). DAPI: 4',6-diamidino-2-phenylindole.

**Figure 2:**
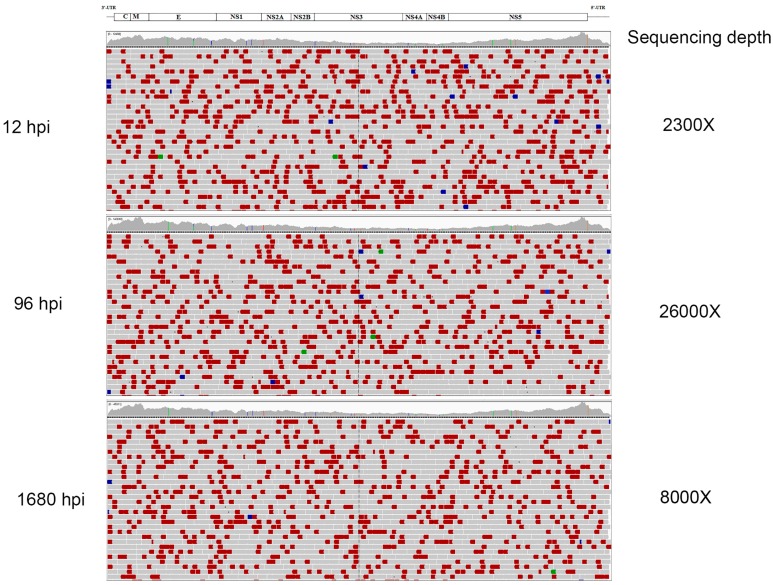
Integrative Genomic Viewer alignment of LGTV TP21 sequence reads obtained at 12, 96 and 1680 hpi. The horizontal gray bars represent sequence read alignments and the colored bars were read pairs of unexpected size or orientation. Genome truncations would have appeared as clear regions interspaced between horizontal gray bars [9]. The LGTV genome map was added as a schematic above the panels.
